# Nicotinamide mononucleotide production by fructophilic lactic acid bacteria

**DOI:** 10.1038/s41598-021-87361-1

**Published:** 2021-04-07

**Authors:** Kazane Sugiyama, Kana Iijima, Miyako Yoshino, Hideo Dohra, Yuji Tokimoto, Koji Nishikawa, Hideaki Idogaki, Nobuyuki Yoshida

**Affiliations:** 1 Department of Engineering, Graduate School of Integrated Science and Technology, Shizuoka University, 3-5-1 Johoku, Naka-ku, Hamamatsu, 432-8561 Japan; 2 Department of Science, Graduate School of Integrated Science and Technology, Shizuoka University, 836 Ohya, Suruga-ku, Shizuoka, 422-8529 Japan; 3 Research Institute of Green Science and Technology, Shizuoka University, Shizuoka, Japan; 4 Osaka Soda Co., Ltd., 12-18 Awaza 1-chome, Nishi-ku, Osaka, 550-0011 Japan

**Keywords:** Applied microbiology, Biotechnology

## Abstract

Nicotinamide mononucleotide (NMN), an intermediate in nicotinamide adenine dinucleotide biosynthesis, is recently attracting much attention for its pharmacological and anti-aging efficacies. However, current commercial products containing NMN are very high-priced because efficient and facile methods for industrial NMN production are limited. In this study, aiming for its nutraceutical application, we attempted to screen lactic acid bacteria for intracellular and/or extracellular NMN production. Using a bioassay system with an auxotrophic yeast that requires nicotinamide riboside (NR; dephosphorylated NMN), three candidates were obtained from a library of 174 strains of facultative anaerobic lactic acid bacteria. All three candidates belonged to the genus *Fructobacillus* and produced NR in the culture media (0.8–1.5 mg/l). Lactic acid bacteria of the genus *Fructobacillus* are known to use d-fructose as an electron acceptor in anaerobic lactic acid fermentation; addition of d-fructose to the medium caused intracellular accumulation of NMN and NR, but no extracellular production of these compounds was observed. Draft genome sequencing for one of the candidates suggested that nicotinamide phosphoribosyltransferase, which exists commonly in mammals but is less reported in microorganisms, is a key enzyme for NMN and NR production in the fructophilic bacteria.

## Introduction

Nicotinamide adenine dinucleotide (NAD^+^) is an important cofactor involved in redox reactions in living systems. NAD^+^ is synthesized from quinolinic acid (QA) generated de novo from l-tryptophan and l-aspartic acid in yeast and bacteria, respectively^1–3^. *Saccharomyces cerevisiae* is known to become a nicotinic acid (NA) auxotroph under anaerobic conditions, and converts NA to nicotinate mononucleotide (NaMN), which is also generated from QA under aerobic conditions^4^. Furthermore, most bacteria have a nicotinamidase that converts nicotinamide (NAM) to NA^5^. Accordingly, microorganisms can also synthesize NAD^+^ by a salvage pathway from NA or NAM incorporated into the cells.

The salvage pathway is predominant in mammalian cells incorporating NAM, nicotinamide mononucleotide (NMN), and nicotinamide riboside (NR) in their diet^6^. NMN is synthesized from NR by a kinase, and from NAM and 5′-phosphoribosyl-1-pyrophosphate (PRPP) by nicotinamide phosphoribosyltransferase (NAMPT)^7^. Subsequently, NAD^+^ is synthesized from NMN by NMN adenylyltransferase. NA and NAM produced by NAD^+^ degradation in cells are also reused in the salvage pathway to resynthesize NAD^+^. Recently, NMN is attracting much attention with increasing evidence for its pharmacological efficacies in several diseases such as myocardial and cerebral ischemia, Alzheimer’s disease, and diabetes mellitus^8,9^. Such pharmacological efficacies involve increased NAD^+^ levels in various tissues and organs^9–16^. Furthermore, NMN is suggested to remedy age-associated decreases in NAD^+^ via NAD^+^ consuming enzymes such as sirtuins, poly ADP-ribose polymerase, and NADase^17^.

Sirtuins (SIRT1-7) are NAD^+^-dependent deacetylases and are known as important regulators of aging and longevity. Increasing SIRT1 in the brain is reported to delay aging and to extend lifespan in both male and female mice^18^ and NMN can restore aging-triggered SIRT1 inactivation^12^. Dysfunction of complex I in the electron transport chain of mitochondria results in NADH accumulation and NAD^+^ deficiency; the subsequent inactivation of mitochondrial SIRT3 causes severe cardiac damage. This was also recovered by supplementation with NMN^11^. Such surprising efficacy of NMN should promote commercial product of NMN-containing products, and some companies do provide some supplements containing NMN. However, commercial NMN-containing products are very expensive (US$ 130–400/g for tablet products; US$ 5–7/g for powder products). Recently, several metabolic engineering approaches were attempted to produce NMN in *Escherichia coli*^19–21^. Marinescu et al. reported NMN production in recombinant *E. coli* carrying genes encoding NAMPT from *Haemophilus ducreyi* and a PRPP synthetase from *Bacillus amyloliquefaciens*^19^. Shoji et al. achieved higher production of NMN by introducing two actively functional transporters (NiaP and PnuC) to the NMN-producing recombinant *E. coli*^20^.

In this study, we focused on lactic acid bacteria (LAB) as NMN-producing microorganisms as LAB cells producing NMN could be used in the industrial NMN production process and the organism itself could be used as probiotics. To screen these LAB, we used a *Saccharomyces cerevisiae* that requires NR for its growth^22^. As the growth of NR-auxotrophic yeast can also be sustained by NMN, we could obtain LAB producing NMN as well as those producing NR. As described above, NR can be converted to NMN in cells, and similar pharmacological effects have been reported for NR as NMN^23,24^.

## Results and discussion

### Screening of NMN (NR)-producing LAB

**Figure 1:**
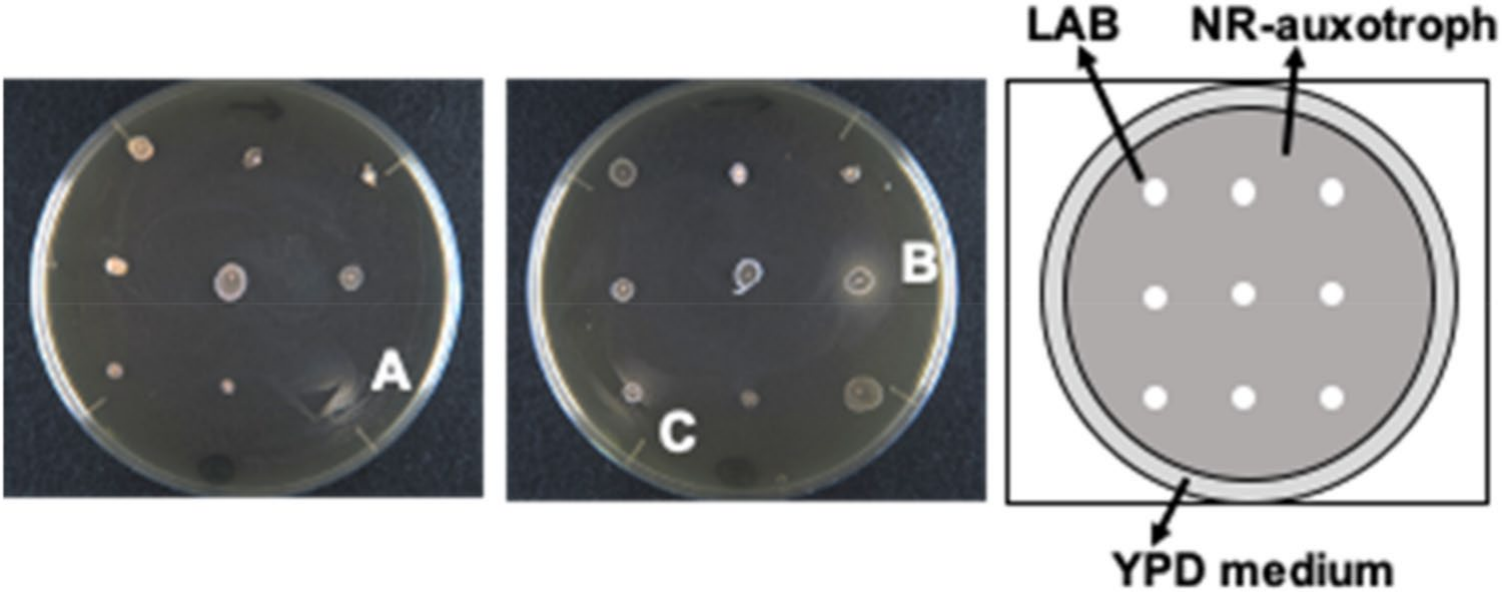
Screening of LAB for their extracellular production of NMN or NR with NR-auxotrophic yeast. LAB cells from their colonies were inoculated onto YPD plates spread with NR-auxotrophic yeast (1.0 × 10^6^ cells) and cultivated at 30 °C for several days. If the LAB produce NMN or NR outside of the cells, a halo of NR-auxotrophic yeast should be observed around the colony of the LAB as shown in the photos (A: RD011727; B: RD012353; C: RD012354).

**Figure 2:**
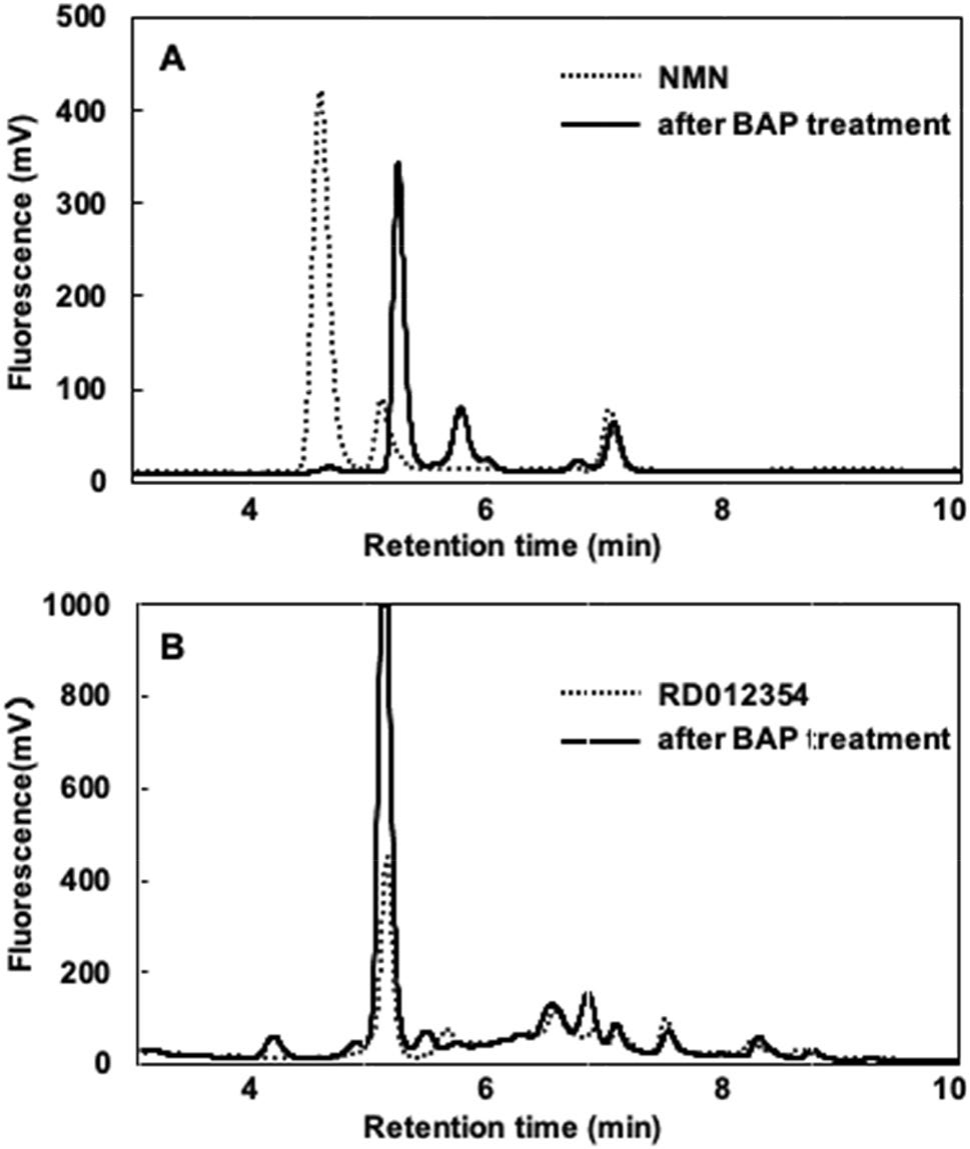
Dephosphorylation of NMN to produce NR by BAP. (**A**) 0.3 nmol of NMN was incubated with 3 U of BAP at 37 °C for 12 h, which underwent fluorescent derivatization, followed by HPLC analysis. (**B**) After 15 ml of the culture filtrate of RD012354 was treated with BAP under the same conditions, NR fraction was extracted with PBA resin and HPLC analysis was performed as described above. Dotted and solid lines represent NR analysis before and after BAP treatment, respectively.

We screened 174 strains of LAB isolated from natural resources (National Institute of Technology and Evaluation, Japan) for their NMN or NR producing activities using an NR-auxotrophic yeast. Three LAB were found to produce halos of the NR-auxotrophic yeast around their colonies, suggesting that these LAB candidates produced and secreted NMN or NR outside their cells (Fig. 1). Analysis of 16S rRNA gene suggested that the three LAB belong to the genus *Fructobacillus*; the closest relative for RD011727 was *Fructobacillus durionis* (99%; accession no. AJ780981), whereas that for RD012353 and RD012354 was *Fructobacillus tropaeoli* (100%; AB542054). Each candidate LAB was cultivated in MRS liquid medium and the amounts of NMN and NR were assessed by high-performance liquid chromatography (HPLC). However, the amounts of the compounds in the culture filtrate of each candidate LAB were below the detection limit on fluorescent HPLC analysis. NR in the culture filtrate could be concentrated by solid phase extraction with phenyl boronate (PBA) resin. Although NMN dissolved in water was extracted with the PBA resin, that in the MRS medium could not be extracted effectively. The candidate LAB were cultivated in the MRS medium for 24 h and the culture filtrate was extracted with PBA resin, followed by fluorescent HPLC. The resulting amounts of NR in the culture filtrates of RD011727, RD012353, and RD012354 were 0.80 ± 0.01, 1.53 ± 0.03, and 0.83 ± 0.01 mg/l, respectively. As a bacterial alkaline phosphatase (BAP) could dephosphorylate NMN to produce NR (Fig. 2A), the culture filtrate was treated with BAP, followed by NR analysis as described above. The resulting peak corresponding to NR in the culture filtrate of RD012354 in HPLC analysis was twice as large as that before BAP treatment (Fig. 2B), suggesting that approximately same amount of NMN as NR existed in the culture filtrate. Analysis with BAP treatment to the culture filtrates from the other LAB showed a similar tendency and the NR peaks in the culture filtrates of RD011727 and RD012353 was increased 2.4 and 1.7 times as large as those before BAP treatment, respectively.

### Effect of fructose addition to the culture medium on NMN and NR production

**Figure 3:**
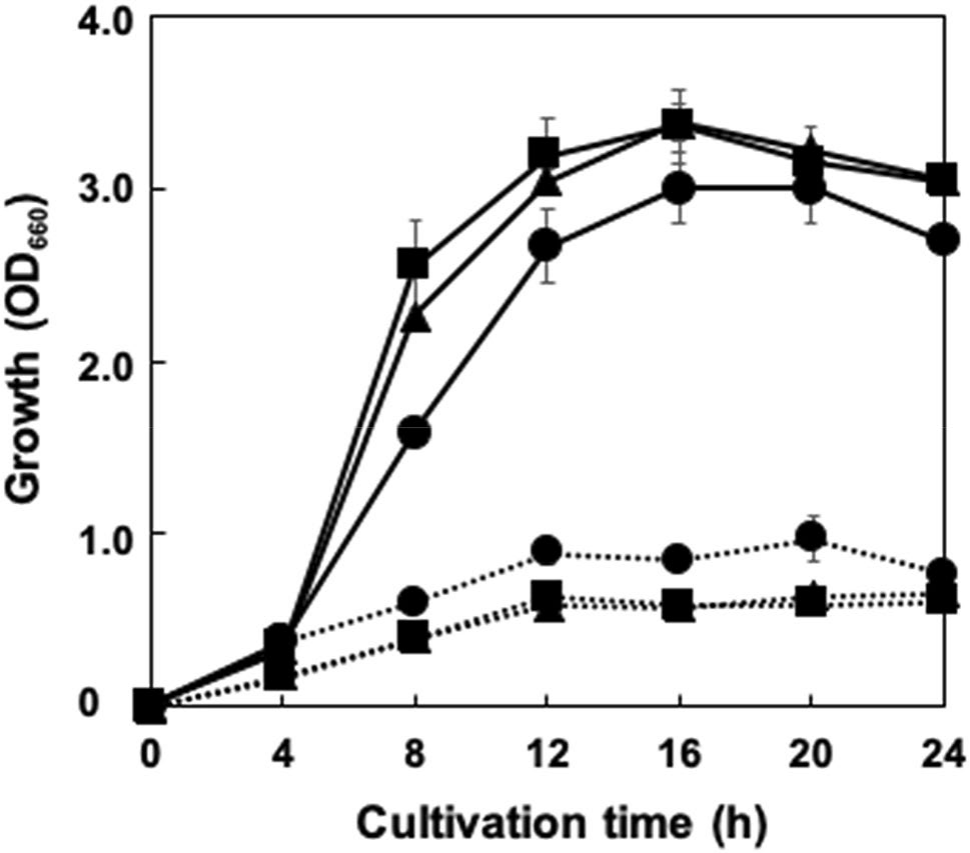
Effect of d-fructose in the culture medium on the growth of the candidate LAB. Each LAB was statically cultivated at 30 °C in 15 ml of MRS medium with (solid lines) or without (dotted lines) d-fructose (1%). Circle, RD011727; triangle, RD012353; square, RD012354. Error bars show standard deviations from biological triplicates.

**Figure 4:**
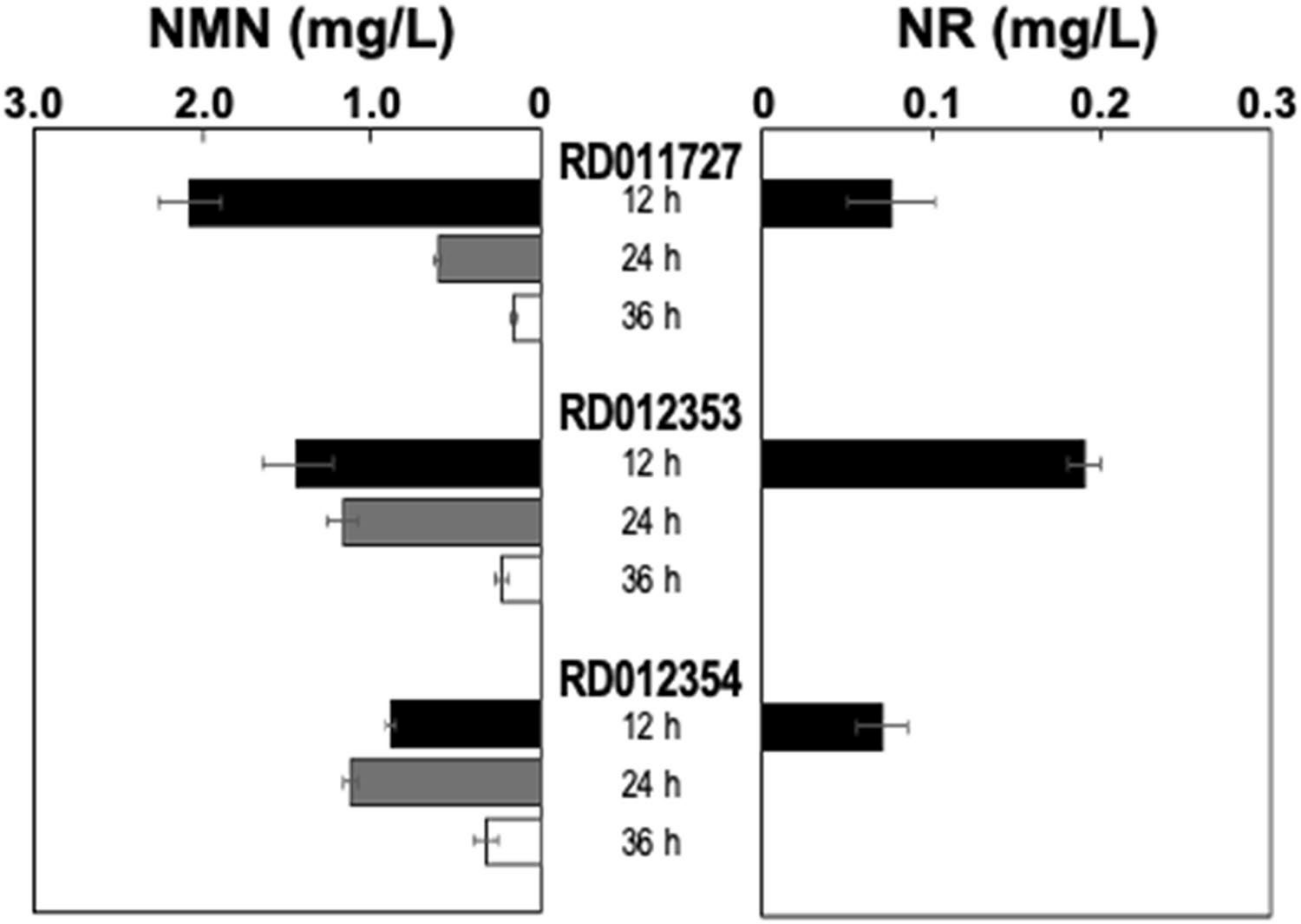
Intracellular accumulation of NMN and NR in the candidate LAB. Each candidate LAB was cultivated in MRS medium containing 1% d-fructose and the amounts of NMN and NR in the cell extract were measured by the fluorescent HPLC analysis. Black, gray, and white bars represent 12-, 24-, and 36-h cultivation, respectively. Error bars show standard deviations from biological triplicates.

As described above, all candidates were identified as LAB belonging to the genus *Fructobacillus* The genus *Fructobacillus* has been recently proposed from the reclassification of genus *Leuconostoc*, and five species have been reported in this genus: *F. fructosus* (type species), *F. durionis*, *F. ficulneus*, *F. pseudoficulneus*, and *F. tropaeoli* (DOI: 10.1099/ijs.0.023838-0)^25^ These *Fructobacillus* spp. are known to produce acetate but not ethanol in lactic heterofermentation as they lack an alcohol/acetaldehyde dehydrogenase gene (*adhE*), and as d-fructose is required as an electron acceptor to maintain the NAD^+^/NADH balance in their cells^26^. Such fermentation characteristics of fructophilic LAB are consistent with the fact that they could be isolated from fructose-rich niches such as flowers and fruits^27^. Considering these findings for fructophilic LAB, the effect of d-fructose addition to MRS medium on NMN and/or NR production was examined in this study, as MRS medium does not contain d-fructose but does contain 1% d-glucose. Addition of d-fructose to MRS medium enhanced the growth of each LAB by approximately 5 times (Fig. 3). Various ratios of d-glucose and d-fructose (1:1, 1:2, 2:1) were examined, but there were no remarkable changes in the growth of each LAB (data not shown). Intriguingly, no extracellular production of NMN and NR was observed in the culture filtrate of each candidate LAB cultivated in fructose-containing MRS medium. This suggested that improved growth by d-fructose addition increased the requirement of NAD^+^ in their cells, which attenuated the leakage of NMN or NR outside the cells. Subsequently, the intracellular amounts of NMN and NR were analyzed for each LAB cultivated in MRS medium containing d-fructose. Figure 4 shows that remarkable NMN production was observed in the cells of each LAB after 12-h cultivation, whereas these amounts were dramatically decreased after 36 h. NR was found only in the 12-h cultivated cells of each candidate, suggesting that NR was produced from an excess intracellular amount of NMN in rapid growing period, whereas it would not be necessary to convert NMN to NR when intracellular amount of NMN was low in the stationary phase. No intracellular accumulation of NMN and NR was observed in each cell grown in the MRS medium without d-fructose.

### Prediction of the NMN-biosynthetic pathway in *Fructobacillus* sp

**Figure 5:**
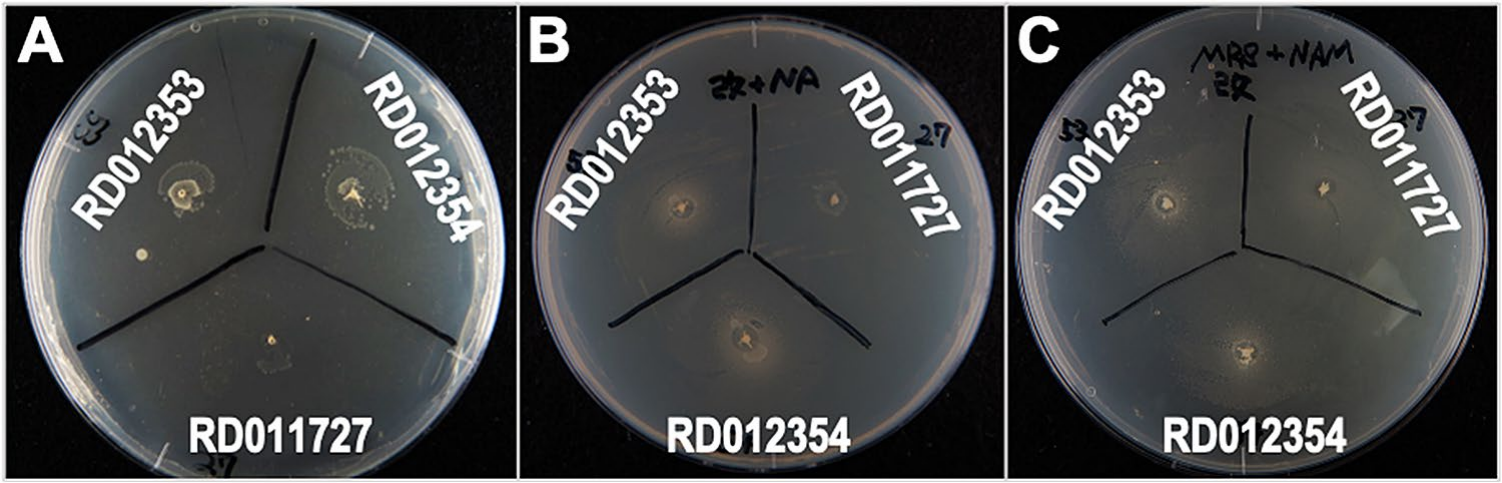
Effect of NA and NAM on the extracellular production of NMN/NR. Each candidate LAB was spotted onto a MRSS plate spread with NR-auxotrophic yeast as described in Fig. 1, and cultivated at 30 °C for 2 days. (**A**) MRSS plate without additive, (**B**) that with 10 µM NA, (**C**) that with 10 µM NAM.

The MRS medium used for NMN (NR) production in this study consists of meat and yeast extracts, which contain various vitamins and minerals as uncertainties. To elucidate the NMN biosynthetic pathway in the candidate LAB, a completely synthetic MRS (MRSS) medium was prepared and used for the NMN (NR) bioassay with the NR-auxotrophic yeast. No growth of the NR-auxotrophic yeast was observed around a colony of each candidate LAB on the MRSS plate (Fig. 5A). However, the NR-auxotrophic yeast was found to grow around RD012353 and RD012354 colonies on MRSS medium containing 10 mM NA, and addition of NAM to the MRSS medium caused yeast growth around the colonies of all the candidate LAB (Fig. 5B,C). These results suggested that the salvage pathway from NA or NAM is involved in the NMN and NR biosynthesis.

**Figure 6:**
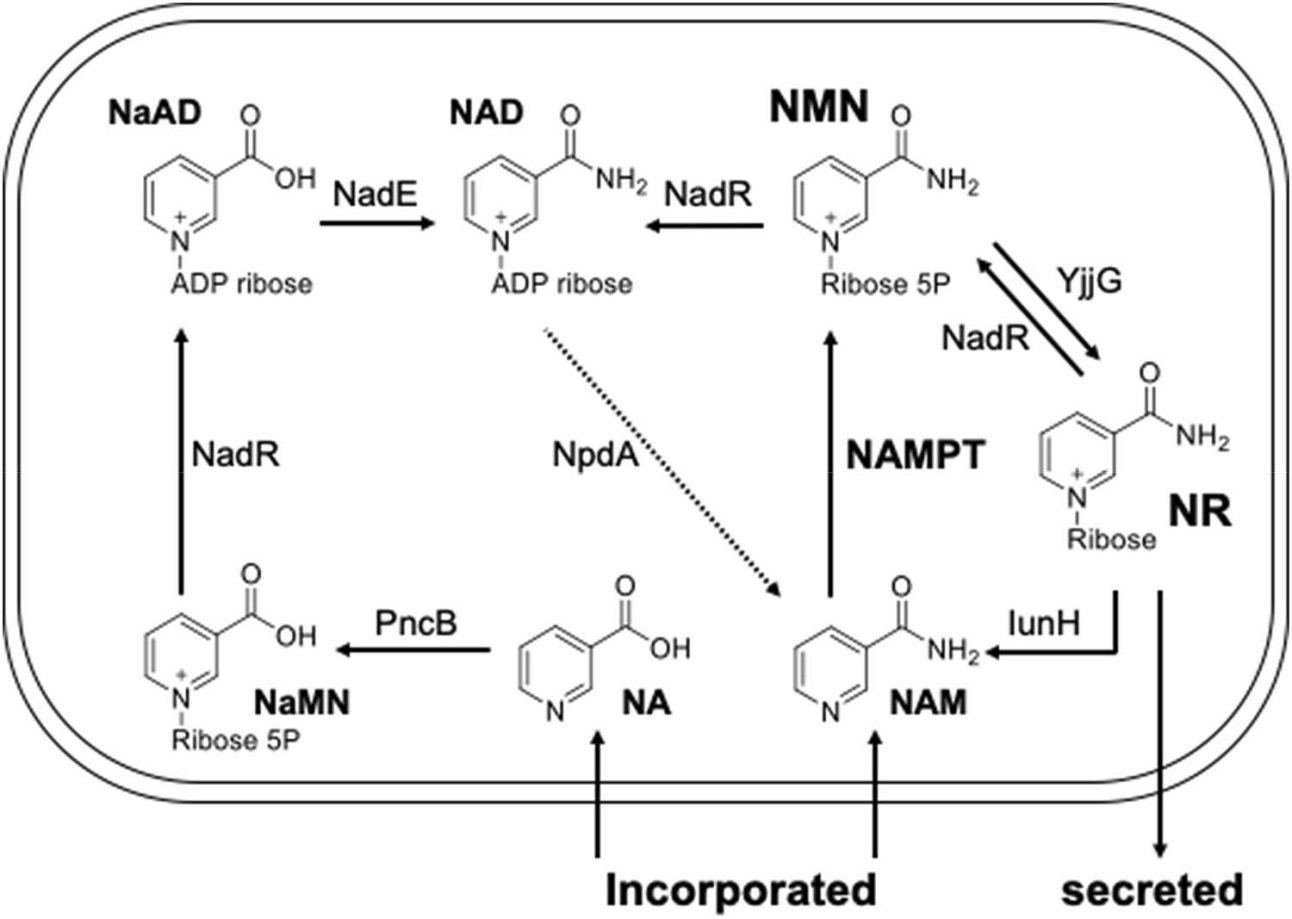
Salvage pathway for NAD^+^ biosynthesis involving NMN and NR in RD012353. *npdA* involved in a pathway indicated by dotted arrow was pseudogenized by a 136-bp deletion in the gene. Enzymes in the figure are as follows: IunH, purine nucleosidase; NadE, NAD synthase; NadR, bifunctional NAD biosynthesis protein; NAMPT, nicotinamide phosphoribosyltransferase; PncB, nicotinate phosphoribosyltransferase; NpdA, NAD-dependent deacetylase; YjjG, 5′-nucleotidase. Compounds in the figure are as follows: NA, nicotinic acid; NaAD, nicotinic acid adenine dinucleotide; NAD, nicotinamide adenine dinucleotide; NAM, nicotinamide; NaMN, nicotinic acid mononucleotide; NMN, nicotinamide mononucleotide; NR, nicotinamide riboside.

Next, we attempted to predict the NMN biosynthetic pathway in one of the candidate LAB, RD012353 by draft genome sequencing, followed by KEGG pathway mapping^28^ and protein family classification using InterProScan^29^. The draft genome sequence of RD012353 consisted of 18 contigs (> 200 base pairs) with a total length of 1,752,368 base pairs, G + C content of 44.0%, *N*_50_ value of 280,510 base pairs, and an average sequence coverage of 175.4×. The genome contains 1621 protein-coding sequences, 6 rRNA genes, and 49 tRNA genes. KEGG pathway mapping showed that seven proteins in RD012353 could be mapped to “Nicotinate and nicotinamide metabolism” (ko00760) (Table S1). Protein functions predicted using KEGG pathway mapping were confirmed by protein family classification and domain prediction using InterProScan (Table S2). Figure 6 shows the salvage pathway for NAD^+^ biosynthesis postulated based on the genome annotation for RD012353. As described above, NMN is synthesized from NAM by NAMPT in mammals, but there are limited reports on microbial NAMPT. Furthermore, this strain does not have a nicotinamidase that catalyzes the conversion of NAM to NA. This postulated metabolic pathway suggests that in RD012353, NMN is produced directly from NAM in culture medium by NAMPT (FT12353_06580). We actually found NAMPT activity (1.52 ± 0.15 mU/mg protein) in the cell-free extract using NAM and PRPP as the substrates and a gene encoding putative PRPP synthetase in the genome of RD012353 (FT12353_15970).

NadR (FT12353_07160) also has an important function related to NAD^+^ as it catalyzes several reactions in the NAD^+^ biosynthetic pathway in RD012353. NadR is annotated as a transcriptional repressor of NAD^+^ biosynthesis genes and is known as a trifunctional protein in *Salmonella enterica*^30^. When NAD^+^ levels are high, NadR binds to NAD^+^, leading to its DNA binding activity that represses several genes involved in de novo NAD^+^ biosynthesis. NadR also has both NMN adenylyltransferase (NMNAT) and nicotinamide ribonucleoside kinase activities. However, NadR of RD012353 lacks a helix-turn-helix DNA binding domain like *Haemophilus influenzae* NadR^31,32^ (Fig. S1), suggesting that the RD012353 NadR is not a transcriptional repressor.

The dotted arrow in Fig. 6 indicates the pathway involving *npdA*, which was not found in initial mapping using KEGG. Sirtuins catalyze a unique protein deacetylation reaction requiring NAD^+^ to produce NAM and *O*-acetyl-ADP ribose. Sirtuin was first identified as a silencer of genes affecting the mating type of yeast, named “silent mating type information regulation 2” (Sir2)^33^. Alignment of the *npdA* genes of *F. tropaeoli* RD012353 and F214-1 revealed that the *npdA* (FT12353_09620) of the RD012353 was pseudogenized by a 136-bp deletion in the gene, suggesting that NAD^+^ is not converted to NAM by NpdA in RD012353. Taken together, these results suggested that in RD012353, NMN is synthesized by NAMPT from NAM incorporated into the cells and is then converted to NAD^+^ by NMNAT activity of NadR under growth enhancing conditions, whereas NMN is dephosphorylated to NR by YjjG (FT12353_00910), which is secreted outside the cells. The reason why fructophilic LAB in this study secreted NR under growth-limiting conditions is unclear, but stagnation of total metabolism under such conditions may give rise to NAD^+^ accumulation, which then causes a disordered cellular redox state due to an unbalanced NAD^+^/NADH ratio. To maintain the NAD^+^/NADH ratio, NMN might convert to NR, which can easily pass through the cell membrane. NpdA is also known as NAD^+^-consuming enzyme to form NAM^8,34^, and the pseudogenization of *npdA* may enhance the reduction of NMN in RD012353 cells.

Although the amount of NMN produced by these recombinant *E. coli* was two or three orders higher than that in our candidate LAB^20^, we are now examining the optimal culture conditions and mutagenesis for our “non-genetic modified” LAB to improve their NMN production. The improved NMN-producing LAB could provide a novel nutraceutical product with anti-aging effect of NMN and probiotic characteristics of LAB.

## Materials and methods

### Microorganisms and culture media

174 strains of LAB were purchase from the National Institute of Technology and Evaluation, Japan. The LAB were statically cultivated in MRS medium (MRS Broth, Becton, Dickinson and Company, Japan). Completely synthetic MRS (MRSS) medium consisted of 1% peptone (Hipolypepton, Nihon Pharmaceutical Co., Ltd., Tokyo), 2% d-glucose, 0.1% Tween 80, 0.2% K_2_HPO_4_, 0.5% sodium acetate, 0.2% diammonium hydrogencitrate, 0.02% MgSO4·7H_2_O, 0.005% MnSO4·4H_2_O, 0.1% trace metal solution (Trace Metal MixA5 with Co, MSD K. K., Tokyo), and 0.0076% uracil.

NR-auxotrophic yeast was kindly gifted from Dr. Su-Ju Lin, UC Davis, and cultivated at 30 °C in YPD medium consisting of 1% yeast extract (BSP-B, Oriental Yeast Co., Ltd.), and 2% d-glucose. When the NR-auxotrophic yeast was cultivated alone, 10 µM NR was added to the YPD medium. Agar (1.5%) was used to prepare a plate of each media.

### Determination of NMN and NR by fluorescent HPLC

Sample (250 µl) containing NMN and/or NR was incubated with 150 µl of 1.3 M KOH and 100 µl of 20% acetophenone at 4 °C for 30 min. Fluorescent derivatization was complete after addition of 400 µl of 98% formic acid, followed by incubation at 110 °C for 7 min. Fifty microliters of the fluorescent derivatives were subjected to HPLC analysis. HPLC analysis was performed using a reverse phase column (Triart C_18_, YMC Co., Ltd., Kyoto) at 30 °C with 0.1% formic acid in water (A) and 0.1% formic acid in acetonitrile (B) as mobile phases in a linear gradient of B (10–70%) for 15 min (1.0 ml/min). The fluorescent derivatives of NMN and NR were detected with an RF-10AXL fluorescent detector (Shimadzu GLC Ltd., Tokyo, Japan) with excitation and emission wavelengths at 320 and 458 nm, respectively.

For the analysis of NMN and NR in the culture filtrate, solid phase extraction with a PBA resin was performed prior to HPLC analysis. Culture filtrate of LAB (15 ml) was mixed with the same volume of 4% NaHCO_3_ was applied on a Bond Elut PBA (100 mg, Agilent Technologies Japan, Ltd.) equilibrated with 2% NaHCO_3_. After washing with 3 ml of 2% NaHCO_3_, the fraction eluted with 3 ml of 2% formic acid was used for the fluorescent derivatization described above.

### Preparation of cell extract and NAMPT assay

Bacterial cells were washed with 0.85% KCl and suspended in 0.1 M potassium phosphate buffer (pH 7.0) containing a protein Inhibitor cocktail (Complete, EDTA-free, Roche Diagnostics K.K., Tokyo,). The cell suspension was disrupted with glass beads and centrifuged at 20,700×*g* for 5 min, and the supernatant was used as a cell extract for intracellular analysis of NMN and NR. The cell extract was also used for measurement of the NAMPT activity as follows.

The reaction mixture containing 100 mM Tris–HCl (pH 7.0), 0.2 mM NAM, 0.2 mM PRPP, 2 mM ATP, 0.25 mM MgCl_2_·6H_2_O, and appropriate amount of the cell extract was incubated at 37 °C for 30 h. The amount of NMN produced by the enzyme reaction was measured by the fluorescent derivatization and the HPLC analysis as described above. One unit of NAMPT activity was defined as an amount of the enzyme that catalyzed 1 µmole of NMN formation per min.

### Draft genome sequencing

The genomic DNA was extracted and purified from RD012353 cells grown in 15 ml of MRS medium containing d-fructose with a MasterPure Gram Positive DNA Purification Kit (epicenter, WI, USA). A library was constructed using the TruSeq Nano DNA Library Prep Kit (Illumina, CA, USA), and the library was sequenced using the Illumina MiSeq platform (301-bp paired-end). The raw reads were cleaned up using Trimmomatic ver. 0.36^35^ by trimming adapter sequences and low-quality reads with the following parameters: CROP:300, SLIDINGWINDOW:4:15, and MINLEN:150. The resultant 628,975 high-quality read pairs totaling 307.3 Mb were assembled using SPAdes ver. 3.13.0^36^ with a default set of *k*-mer sizes and options “-careful and -cov-cutoff auto”. The draft genome sequence of RD012353 was annotated using DFAST-core ver. 1.2.6^37^ with an in-house database created from 10 genome information of the genus *Fructobacillus* deposited in the NCBI RefSeq database (July 1, 2020), and the annotations were manually curated. The predicted gene products were also annotated using KofamKOALA ver. 2020-06-07^38^ and InterProScan ver. 5.45-80.0^26^ to find candidate enzymes involved in the NMN biosynthetic pathway.

### Ethical standards

This article does not contain any studies with human participants performed by any of the authors.

## Supplementary Information

Supplementary Information 1.

## Data Availability

The raw read sequences of *F. tropaeoli* RD012353 were deposited in the DDBJ Sequence Read Archive (DRA) under the accession no. DRR237061. This whole genome shotgun sequencing project has been deposited in the DDBJ/ENA/GenBank under the accession no. BOJU00000000.
