# Supplementary material for: Nicotinamide mononucleotide production by fructophilic lactic acid bacteria
Source: Sci Rep. 2021 Apr 7;11:7662. doi: 10.1038/s41598-021-87361-1 (PMC8027369; doi:10.1038/s41598-021-87361-1)
Supplement: Supplementary file 1 — Supplementary Information 1. [file 41598_2021_87361_MOESM1_ESM.docx]

**Supplementary Information**

Title: **Nicotinamide mononucleotide production by fructophilic lactic acid bacteria**

Authors: Kazane Sugiyama^1^, Kana Iijima^1^, Miyako Yoshino^1^, Hideo Dohra^2, 3^, Yuji Tokimoto^4^, Koji Nishikawa^4^, Hideaki Idogaki^4^, and Nobuyuki Yoshida^1^*

^1^Department of Engineering, Graduate School of Integrated Science and Technology, Shizuoka University, 3-5-1 Johoku, Naka-ku, Hamamatsu 432-8561, Japan; ^2^Department of Science, Graduate School of Integrated Science and Technology, Shizuoka University, 836 Ohya, Suruga-ku, Shizuoka 422-8529, Japan; ^3^Research Institute of Green Science and Technology, Shizuoka University, Shizuoka, Japan; ^4^Osaka Soda Co., Ltd., 12-18 Awaza 1-chome, Nishi-ku, Osaka 550-0011, Japan

**This file includes:**

**•Fig. S1**

**•Table S1 and S2**

**
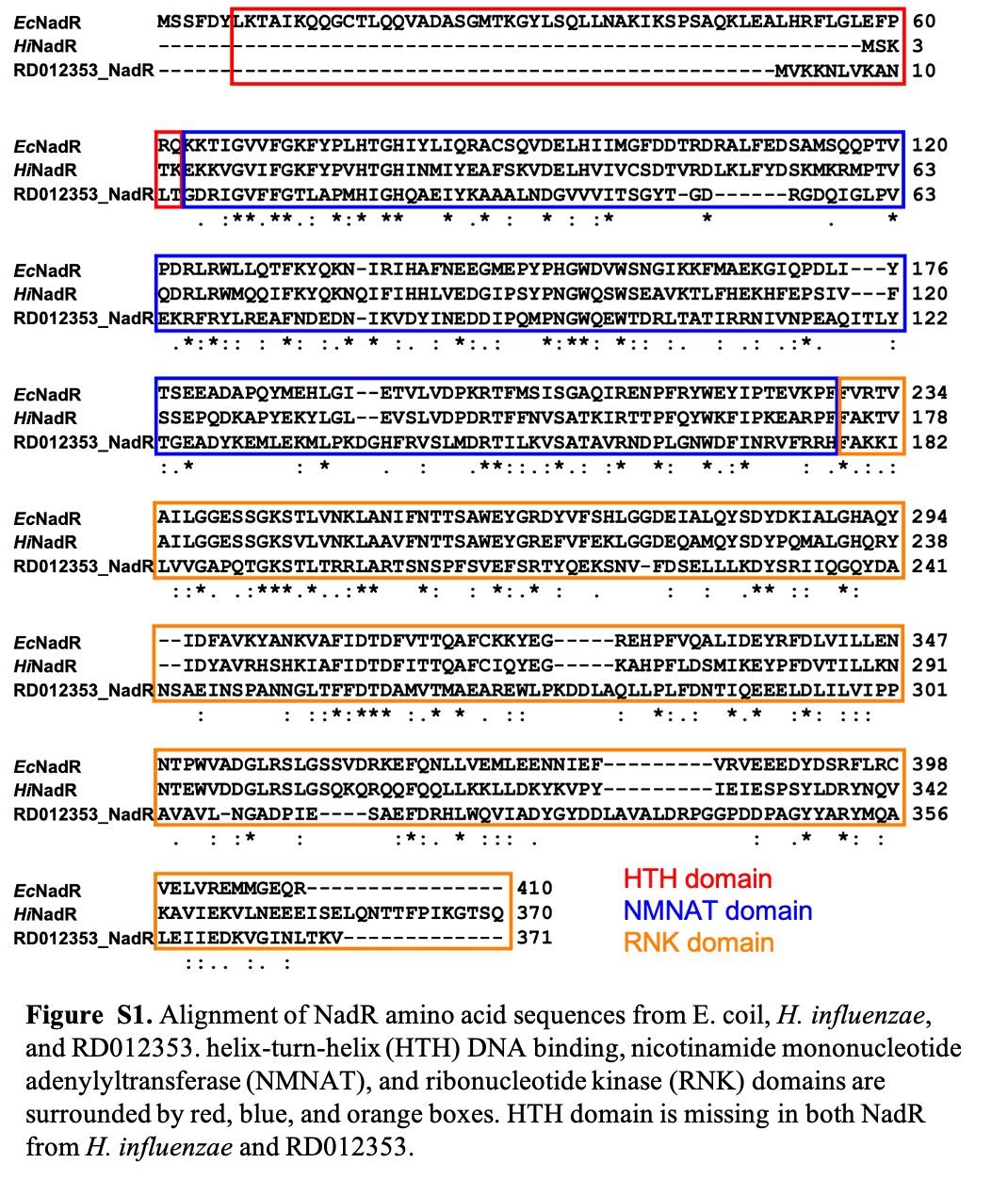
**

| **Table S1. KEGG orthology assignments of enzymes mapped to "Nicotinate and nicotinamide metabolism" (ko00760) by KofamKOALA** | | | |
| --- | --- | --- | --- |
| Locus tag | KEGG orthology | Name | Definition |
| FT12353_00910 * | K08723 | yjjG | 5'-nucleotidase [EC:3.1.3.5] |
| FT12353_03760 | K00858 | ppnK,  NADK | NAD+ kinase [EC:2.7.1.23] |
| FT12353_06580 | K03462 | NAMPT | nicotinamide phosphoribosyltransferase [EC:2.4.2.12] |
| FT12353_07160 | K06211 | nadR | HTH-type transcriptional regulator, transcriptional repressor of NAD biosynthesis genes [EC:2.7.7.1 2.7.1.22] |
| FT12353_09620 (pseudogene) ** | K12410 | npdA | NAD-dependent deacetylase [EC:2.3.1.286] |
| FT12353_14900 | K01916 | nadE | NAD+ synthase [EC:6.3.1.5] |
| FT12353_14910 | K00763 | pncB | nicotinate phosphoribosyltransferase [EC:6.3.4.21] |
| FT12353_15920 | K01239 | iunH | purine nucleosidase [EC:3.2.2.1] |
| * FT12353_00910 was not assigned to K08723 by the initial mapping because of the score lower than the threshold, but the score was the highest for K08723. | | | |
| ** Orthologous protein (*Fructobacillus tropaeoli* F214-1, WP_059393821.1) of pseudogenized FT12353_09620 was assigned to K12410. | | | |

| **Table S2. Protein family classification and domain prediction of enzymes mapped to "Nicotinate and nicotinamide metabolism" (ko00760) by InterProScan** | | | | | |
| --- | --- | --- | --- | --- | --- |
| Locus tag | Database | ID | Name | InterPro ID | InterPro name |
| FT12353_00910 | Gene3D | G3DSA:1.10.150.240 | Putative phosphatase; domain 2 | IPR023198 | Phosphoglycolate phosphatase-like, domain 2 |
| FT12353_00910 | Gene3D | G3DSA:3.40.50.1000 | - | IPR023214 | HAD superfamily |
| FT12353_00910 | PANTHER | PTHR47478 | - | - | - |
| FT12353_00910 | PANTHER | PTHR47478:SF1 | PYRIMIDINE 5'-NUCLEOTIDASE YJJG | - | - |
| FT12353_00910 | Pfam | PF13419 | Haloacid dehalogenase-like hydrolase | IPR041492 | Haloacid dehalogenase-like hydrolase |
| FT12353_00910 | SFLD | SFLDG01129 | C1.5: HAD, Beta-PGM, Phosphatase Like | - | - |
| FT12353_00910 | SFLD | SFLDS00003 | Haloacid Dehalogenase | - | - |
| FT12353_00910 | SUPERFAMILY | SSF56784 | HAD-like | IPR036412 | HAD-like superfamily |
| FT12353_00910 | TIGRFAM | TIGR01549 | HAD-SF-IA-v1: HAD hydrolase, family IA, variant 1 | IPR006439 | HAD hydrolase, subfamily IA |
| FT12353_00910 | TIGRFAM | TIGR02254 | YjjG/YfnB: noncanonical pyrimidine nucleotidase, YjjG family | IPR011951 | HAD-superfamily hydrolase, subfamily IA, YjjG/YfnB |
| FT12353_03760 | Gene3D | G3DSA:2.60.200.30 | - | IPR017437 | ATP-NAD kinase, PpnK-type, C-terminal |
| FT12353_03760 | Gene3D | G3DSA:3.40.50.10330 | - | IPR017438 | Inorganic polyphosphate/ATP-NAD kinase, N-terminal |
| FT12353_03760 | Hamap | MF_00361 | NAD kinase [nadK]. | IPR002504 | NAD kinase |

| **Table S2 (continued)** | |  |  |  |  |
| --- | --- | --- | --- | --- | --- |
| Locus tag | Database | ID | Name | InterPro ID | InterPro name |
| FT12353_03760 | PANTHER | PTHR20275 | - | - | - |
| FT12353_03760 | PANTHER | PTHR20275:SF9 | - | - | - |
| FT12353_03760 | Pfam | PF01513 | ATP-NAD kinase | IPR002504 | NAD kinase |
| FT12353_03760 | SUPERFAMILY | SSF111331 | NAD kinase/diacylglycerol kinase-like | IPR016064 | NAD kinase/diacylglycerol kinase-like domain superfamily |
| FT12353_06580 | Gene3D | G3DSA:3.20.20.70 | Aldolase class I | IPR013785 | Aldolase-type TIM barrel |
| FT12353_06580 | PANTHER | PTHR43816 | - | IPR016471 | Nicotinamide phosphoribosyl transferase |
| FT12353_06580 | PANTHER | PTHR43816:SF1 | NICOTINAMIDE PHOSPHORIBOSYLTRANSFERASE | - | - |
| FT12353_06580 | Pfam | PF04095 | Nicotinate phosphoribosyltransferase (NAPRTase) family | IPR041525 | Nicotinate/nicotinamide phosphoribosyltransferase |
| FT12353_06580 | Pfam | PF18127 | Domain of unknown function (DUF5598) | IPR041529 | Nicotinamide phosphoribosyltransferase, N-terminal domain |
| FT12353_06580 | PIRSF | PIRSF005943 | NMPRT | IPR016471 | Nicotinamide phosphoribosyl transferase |
| FT12353_06580 | SUPERFAMILY | SSF51690 | Nicotinate/Quinolinate PRTase C-terminal domain-like | IPR036068 | Nicotinate phosphoribosyltransferase-like, C-terminal |
| FT12353_07160 | Gene3D | G3DSA:3.40.50.300 | - | - | - |

| **Table S2 (continued)** | |  |  |  |  |
| --- | --- | --- | --- | --- | --- |
| Locus tag | Database | ID | Name | InterPro ID | InterPro name |
| FT12353_07160 | Gene3D | G3DSA:3.40.50.620 | HUPs | IPR014729 | Rossmann-like alpha/beta/alpha sandwich fold |
| FT12353_07160 | PANTHER | PTHR37512 | - | - | - |
| FT12353_07160 | Pfam | PF13521 | AAA domain | IPR038727 | NadR/Ttd14, AAA domain |
| FT12353_07160 | PIRSF | PIRSF004776 | NadR_NMNAT/RNK | IPR016429 | NAD biosynthesis/regulator protein NadR |
| FT12353_07160 | SUPERFAMILY | SSF52374 | Nucleotidylyl transferase | - | - |
| FT12353_07160 | SUPERFAMILY | SSF52540 | P-loop containing nucleoside triphosphate hydrolases | IPR027417 | P-loop containing nucleoside triphosphate hydrolase |
| FT12353_09620 (pseudogene) | Gene3D | G3DSA:3.30.1600.10 | SIR2/SIRT2 'Small Domain' | IPR026591 | Sirtuin, catalytic core small domain superfamily |
| FT12353_09620 (pseudogene) | Gene3D | G3DSA:3.40.50.1220 | - | - | - |
| FT12353_09620 (pseudogene) | PANTHER | PTHR11085 | HISTONE DEACETYLASE SIR2 FAMILY MEMBER | - | - |
| FT12353_09620 (pseudogene) | PANTHER | PTHR11085:SF15 | NAD-DEPENDENT PROTEIN DEACETYLASE SIRTUIN-1 | - | - |
| FT12353_09620 (pseudogene) | Pfam | PF02146 | Sir2 family | IPR003000 | Sirtuin family |
| FT12353_09620 (pseudogene) | ProSiteProfiles | PS50305 | Sirtuin catalytic domain profile. | IPR026590 | Sirtuin family, catalytic core domain |

| **Table S2 (continued)** | |  |  |  |  |
| --- | --- | --- | --- | --- | --- |
| Locus tag | Database | ID | Name | InterPro ID | InterPro name |
| FT12353_09620 (pseudogene) | SUPERFAMILY | SSF52467 | DHS-like NAD/FAD-binding domain | IPR029035 | DHS-like NAD/FAD-binding domain superfamily |
| FT12353_14900 | CDD | cd00553 | NAD_synthase | IPR003694 | NAD(+) synthetase |
| FT12353_14900 | Gene3D | G3DSA:3.40.50.620 | HUPs | IPR014729 | Rossmann-like alpha/beta/alpha sandwich fold |
| FT12353_14900 | Hamap | MF_00193 | NH(3)-dependent NAD(+) synthetase [nadE]. | IPR022926 | NH(3)-dependent NAD(+) synthetase |
| FT12353_14900 | PANTHER | PTHR23090 | NH 3 /GLUTAMINE-DEPENDENT NAD + SYNTHETASE | IPR003694 | NAD(+) synthetase |
| FT12353_14900 | PANTHER | PTHR23090:SF7 | NH(3)-DEPENDENT NAD(+) SYNTHETASE | - | - |
| FT12353_14900 | Pfam | PF02540 | NAD synthase | IPR022310 | NAD/GMP synthase |
| FT12353_14900 | SUPERFAMILY | SSF52402 | Adenine nucleotide alpha hydrolases-like | - | - |
| FT12353_14900 | TIGRFAM | TIGR00552 | nadE: NAD+ synthetase | IPR003694 | NAD(+) synthetase |
| FT12353_14910 | CDD | cd01570 | NAPRTase_A | - | - |
| FT12353_14910 | Gene3D | G3DSA:3.20.140.10 | nicotinate phosphoribosyltransferase | - | - |
| FT12353_14910 | Gene3D | G3DSA:3.20.20.70 | Aldolase class I | IPR013785 | Aldolase-type TIM barrel |

| **Table S2 (continued)** | |  |  |  |  |
| --- | --- | --- | --- | --- | --- |
| Locus tag | Database | ID | Name | InterPro ID | InterPro name |
| FT12353_14910 | PANTHER | PTHR11098 | NICOTINATE PHOSPHORIBOSYLTRANSFERASE | IPR007229 | Nicotinate phosphoribosyltransferase family |
| FT12353_14910 | PANTHER | PTHR11098:SF1 | NICOTINATE PHOSPHORIBOSYLTRANSFERASE | - | - |
| FT12353_14910 | Pfam | PF04095 | Nicotinate phosphoribosyltransferase (NAPRTase) family | IPR041525 | Nicotinate/nicotinamide phosphoribosyltransferase |
| FT12353_14910 | Pfam | PF17767 | Nicotinate phosphoribosyltransferase (NAPRTase) N-terminal domain | IPR040727 | Nicotinate phosphoribosyltransferase, N-terminal domain |
| FT12353_14910 | Pfam | PF17956 | Nicotinate phosphoribosyltransferase C-terminal domain | IPR041619 | Nicotinate phosphoribosyltransferase C-terminal domain |
| FT12353_14910 | PIRSF | PIRSF000484 | NAPRT | IPR007229 | Nicotinate phosphoribosyltransferase family |
| FT12353_14910 | SUPERFAMILY | SSF51690 | Nicotinate/Quinolinate PRTase C-terminal domain-like | IPR036068 | Nicotinate phosphoribosyltransferase-like, C-terminal |

| **Table S2 (continued)** | |  |  |  |  |
| --- | --- | --- | --- | --- | --- |
| Locus tag | Database | ID | Name | InterPro ID | InterPro name |
| FT12353_14910 | SUPERFAMILY | SSF54675 | Nicotinate/Quinolinate PRTase N-terminal domain-like | - | - |
| FT12353_14910 | TIGRFAM | TIGR01513 | NAPRTase_put: nicotinate phosphoribosyltransferase | IPR006405 | Nicotinate phosphoribosyltransferase pncB-type |
| FT12353_15920 | CDD | cd02650 | nuc_hydro_CaPnhB | - | - |
| FT12353_15920 | Gene3D | G3DSA:3.90.245.10 | - | IPR036452 | Ribonucleoside hydrolase-like |
| FT12353_15920 | PANTHER | PTHR12304 | INOSINE-URIDINE PREFERRING NUCLEOSIDE HYDROLASE | IPR023186 | Inosine/uridine-preferring nucleoside hydrolase |
| FT12353_15920 | PANTHER | PTHR12304:SF48 | PYRIMIDINE-SPECIFIC RIBONUCLEOSIDE HYDROLASE RIHA-RELATED | - | - |
| FT12353_15920 | Pfam | PF01156 | Inosine-uridine preferring nucleoside hydrolase | IPR001910 | Inosine/uridine-preferring nucleoside hydrolase domain |
| FT12353_15920 | SUPERFAMILY | SSF53590 | Nucleoside hydrolase | IPR036452 | Ribonucleoside hydrolase-like |
